# Evaluation of a Carepartner-Integrated Telehealth Gait Rehabilitation Program for Persons with Stroke : Study Protocol for a Feasibility Study

**DOI:** 10.21203/rs.3.rs-2689016/v1

**Published:** 2023-04-14

**Authors:** Sarah Blanton, George Cotsonis, Kayla Brenan, Robert Song, Laura Zajac-Cox, Sarah Caston, Heather Stewart, Arun Jayaraman, Darcy Reisman, Patricia C Clark, Trisha Kesar

**Affiliations:** Emory University; Emory University School of Public Health; Emory University Hospital; Emory University School of Medicine; Emory University School of Medicine; Emory University School of Medicine; Emory University Hospital; Northwestern University Department of Physical Medicine and Rehabilitation; University of Delaware Department of Physical Therapy; Georgia State University Byrdine F Lewis School of Nursing and Health Professions; Emory University School of Medicine

**Keywords:** Caregiver, Stroke, Rehabilitation, Telehealth, Gait, Dyads

## Abstract

**Background:**

Despite family carepartners of individuals post-stroke experiencing high levels of strain and reduced quality of life, stroke rehabilitation interventions rarely address carepartner well-being or offer training to support their engagement in therapeutic activities. Our group has developed creative intervention approaches to support families during stroke recovery, thereby improving physical and psychosocial outcomes for both carepartners and stroke survivors. The purpose of this preliminary clinical trial is to test the feasibility of an adapted, home-based intervention (Carepartner Collaborative Integrative Therapy for Gait-CARE-CITE-Gait) designed to facilitate positive carepartner involvement during home-based training targeting gait and mobility.

**Methods:**

This two-phased study will determine the feasibility of CARE-CITE-Gait, a novel intervention developed by our team that leverages principles from our previous carepartner-focused upper extremity intervention. During the 4-week CARE-CITE-Gait intervention, carepartners review online video-based modules designed to illustrate strategies for an autonomy-supportive environment during functional mobility task practice, and the study team completes two 2-hour (home-based) visits for dyad collaborative goal setting. In Phase I, the usability and acceptability of the CARE-CITE-Gait modules will be evaluated by stroke rehabilitation content experts and carepartners. In Phase II, feasibility (based on measures of recruitment, retention, and intervention adherence) will be measured. Preliminary effects of the CARE-CITE-Gait will be gathered using a single-group, evaluator blinded, quasi-experimental design with repeated measures (two baseline visits one week apart, post-test, and one-month follow-up) with 15 carepartner and stroke survivor dyads. Outcomes include psychosocial variables (strain, family conflict surrounding stroke recovery, autonomy support and life changes) collected from carepartners, and measures of functional mobility, gait speed, stepping activity, and health-related quality of life collected from stroke survivors.

**Discussion:**

The findings of the feasibility testing and preliminary data on the effects of CARE-CITE-Gait will provide justification and information to guide a future definitive randomized clinical trial. The knowledge gained from this study will enhance our understanding of and aid the development of rehabilitation approaches that address both carepartner and stroke survivor needs during the stroke recovery process.

**Trial Registration:**

ClinicalTrials.gov, NCT 05257928. Registered 25 February 2022, https://clinicaltrials.gov/ct2/show/NCT05257928

## Background

Recognized as an independent risk factor for other co-morbidities and a recurrent stroke,[1] reduced physical inactivity in stroke survivors (SS) is a critical area of rehabilitation focus.[2] Even after discharge from rehabilitation, a large proportion of stroke survivors continue to have persistent impairments in gait and balance, which in turn reduce community ambulation and adversely affect quality of life. Few SS meet the current American Stroke Association guidelines[3] of 20–60 minutes of aerobic exercise 3 to 5 days per week. Typically, SS achieve only 63% of the recommended steps per day for people with disability (4078 versus 6500–8500).[4] Fini et., al.[2] cite motivation and carepartner (CP) support as potential factors to consider, underscoring self-management and behavior change to enhance engagement and sustainability of post-stroke physical activity programs. However, lacking training and preparation to support the complexities of stroke rehabilitation,[5–7] CPs can experience high levels of care burden, reduced quality of life, and family conflict surrounding the post-stroke recovery process [8,9].

Because the physical, emotional, and psychological aspects of their daily living are intricately interconnected, CP’s well-being can impact the health outcomes of both CPs and SSs. With earlier discharge and a greater proportion of rehabilitation occurring at home, it is critical to find strategies to enable CPs to support rehabilitation at home and in the community without adding to their own caregiving burden. Previous research studies incorporating CP training during SS rehabilitation have had promising results. [10, 11] The London Stroke Carers Training Course, a comprehensive competency training for CPs, reduced health and social care costs, improved QOL, and reduced CP burden, [11] but these findings were not replicated in a multi-site study.[12] Work by Creasy et. al.[13] suggests that CPs are keenly aware of the critical nature of their role in the care of their loved one and that they have expectations of being included in post-stroke treatment planning. CPs underscored their need to have greater information about stroke and customization of rehabilitation training to more accurately address specific family needs. [14] Several studies have shown that improving CP coping and life skills to care for a chronically ill family member leads to decreased caregiver burden and improved QOL[15, 16] [17]. Supporting previous summary recommendations [7, 17], a recent systematic review of stroke family CP and dyad interventions[18] emphasized that these interventions should combine skill building (e.g., problem-solving, goal setting, and stress management) with psychoeducational strategies and should be tailored to individualized needs of the CP. Engagement of CPs offers valuable opportunities to enhance rehabilitation therapies.

If therapeutic interventions are delivered in a vacuum without consideration of family context, a fundamental cornerstone influencing successful stroke recovery is overlooked. To address this critical gap, our team developed a theory-based, task-specific, and CP-focused intervention - **Care**partner and **C**ollaborative **I**ntegrated **T**h**e**rapy (**CARE-CITE**)[19], The initial version of CARE-CITE was designed to enhance upper extremity (UE) functional task practice in the home setting by instructing CPs in methods for collaborative goal setting and creating an autonomy-supportive environment. Arising from Self-Determination Theory[20], CARE-CITE uses web-based interactive modules with exemplary videos of actual stroke dyads (CP and SS) demonstrating collaborative problem-solving during activities of daily living and modeling autonomy-supportive dialogue that offers choice, rationale, providing empathy, and avoids controlling language. Preliminary studies show the feasibility[21] and promising therapeutic benefits of CARE-CITE in CPs and SS coupled with UE constraint-induced movement therapy (CIMT), another evidence-supported UE intervention. In a two-group randomized pilot study (n = 32 dyads), we compared a CARE-CITE with CIMT group to a CIMT-alone group of SS who had completed formal rehabilitation therapy.[22] Both groups received 10 sessions of home-based CIMT, but only CPs in the CARE-CITE with CIMT group reviewed the online CARE-CITE modules. Importantly, in our baseline data with these SSs in the chronic phase of stroke, [22] we found that the majority of CPs continue to experience family conflict surrounding stroke recovery, potentially influencing SS motivation and adherence. Higher levels of conflict around stroke recovery were related to higher levels of CP strain and less autonomy support provided to the SS for rehabilitation activities. Outcomes from the study showed promising trends indicating CP’s receiving CARE-CITE had improved psychosocial outcomes (significant within-group CP changes were found for CIMT + CARE-CITE but not CIMT-alone), showed positive quality of life changes, with less strain, fatigue, and family conflict around stroke recovery. [23,24] In both groups SS upper extremity (UE) function and health-related quality of life (hrQOL) improved.[24] However, only the CARE-CITE + CIMT group maintained or continued to improve UE function. These results suggest that CP engagement may be an important factor in the continued support of functional progress in rehabilitation.

The COVID-19 pandemic has revealed both an urgent need and opportunity to craft multiple modes of delivery for interventions for families after stroke, in particular the development of telerehabilitation approaches.[18, 25] Web-based approaches like CARE-CITE-Gait increase accessibility to content outside of traditional clinic hours and reduce transportation-related barriers. While many CP may still lack resources and skills to use web-based approaches, the pandemic has rapidly increased the adoption of these technologies and a recent study [26] found that 86.1 % of SS and CP had internet access. Implementing telerehabilitation delivery of CP interventions offers promising and scalable alternatives to improve access to care.

Responding to the insights gained from the engagement of the CP in UE rehabilitation and the increasing need for telerehabilitation, we now seek to broaden the scope of our intervention and further optimize post-stroke recovery by pairing CARE-CITE with home-based gait and functional mobility training (CARE-CITE-Gait). Physical inactivity resulting after stroke has been associated with physical and psychosocial problems,[27] including increased risk of recurrent stroke. Despite being a critical and modifiable risk factor,[1] most SS do not engage in enough physical activity to meet the American Stroke Association guidelines.[3] In addition to physical impairments (such as balance and weakness) impacting physical activity, other important factors include SS mood, motivation, and CP involvement. [2] Designing interventions that support physical activity by engaging family CPs may be a key factor for the sustainability of health-related behaviors.

In the current study, we are modifying our existing CARE-CITE intervention to address gait and functional mobility, and test 4 weeks of CARE-CITE-Gait in 15 CP and SS dyads. The aim of this paper is to describe the CARE-CITE-Gait, two-phased, feasibility study design. The objective of Phase I of this trial is to evaluate the content validity and user satisfaction (usability and acceptability) of the CARE-CITE-Gait intervention by CPs and stroke rehabilitation content experts. The primary objective of Phase II is to determine the feasibility of CARE-CITE-Gait for SS and CP dyads, as indicated by participant recruitment and retention, SS and CP adherence to the intervention, CP usability and satisfaction with the CARE-CITE-Gait modules, and safety (occurrence of SS adverse events). The secondary objective of Phase II is to determine the preliminary effects of the CARE-CITE-Gait intervention on SS and CPs. Psychosocial outcomes will be measured for the CP, including family conflict about stroke recovery, strain, autonomy support, and positive life changes. Outcomes measured from the SS include functional mobility, gait speed, stepping activity, and health-related quality of life (hrQOL).

## Methods

Identification and reporting of relevant elements of this protocol are based on the Standard Protocol Items: Recommendations for Intervention Trials (SPIRIT) checklist [28] and Template for Intervention Description and Replication (TIDieR) guidelines for intervention descriptions [29]. This is the first published version of this clinical trial study protocol [11.17.2018]. Any protocol amendments will be immediately reported to University Internal Review Board for approval and to funding agency as appropriate.

### Approvals

Ethical approval was obtained by Emory University Institutional Review Board and this protocol is registered on clinicaltrials.gov (NCT05257928).

### Study Design & Setting

**Figure 1:**
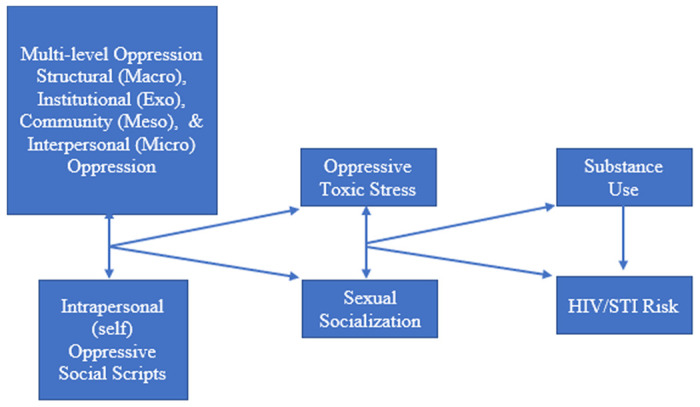
Consort Flow Chart: Evaluation of a Carepartner-Integrated Telehealth Gait Rehabilitation Program for Persons with Stroke (CARE-CITE-Gait) (N=5). [see attachment]

In Phase I, stroke rehabilitation content experts and CPs will evaluate the usability and acceptability of the CARE-CITE-Gait modules. In Phase II, feasibility (based on measures of recruitment, retention, and intervention adherence) and preliminary effects of CARE-CITE-Gait will be measured using a one-group, quasi-experimental design with repeated measures (two baseline visits one week apart, post-test, and one-month follow-up) with 15 CP and SS dyads. While the in-person portion of the intervention will take place in participant homes, the study recruitment, screening, and evaluations (evaluator-blinded) for CP and SS dyads, will occur in a stroke research laboratory within an urban rehabilitation hospital located in Atlanta, Georgia, United States (Figure 1 Consort Flow Chart: Evaluation of Carepartner-Integrated Telehealth Gait Rehabilitation Program for Persons with Stroke (CARE-CITE-Gait) N=15)). Licensed physical therapists will conduct the evaluations and administer the 4-week home-based CARE-CITE-Gait intervention. During the intervention period, the web-based CARE-CITE-Gait modules will be accessed online by CPs independent of therapist involvement. The study schedule schematic design is presented in Table 1.

### Recruitment

#### Participants

Expanding on the established validity and satisfaction demonstrated for the original upper extremity CARE-CITE intervention [21], for Phase I of the study, six to seven stroke rehabilitation clinicians and research content experts will evaluate the content validity of CARE-CITE-Gait adaption of the upper extremity functional tasks to gait and mobility focused examples. Three CPs will provide initial feedback regarding satisfaction (usefulness, ease of use, and acceptability) to guide any additional refinements before the initiation of Phase II.

For Phase II of this study, 15 SS and CP dyads will be recruited. Both CP and SS must be greater than 21 years of age, able to read and write English, and able to provide informed consent.

Inclusion criteria for the SS include:

- > 3 months post ischemic or hemorrhagic stroke event
- Discharged from inpatient neurologic rehabilitation to their home
- Able to walk 10-meters with or without an assistive device
- Presence of CP

Exclusion criteria for SS include:

- Severe cognitive deficits (as indicated by Mini-mental test ≤ 24)
- Concurrent participation in other rehabilitation intervention research trials
- Physician determined major medical or musculoskeletal problems that would limit study participation

CPs will be self-identified as a spouse/partner or family member dwelling in the same household who has the role as the primary caregiver of the SS. The primary CP inclusion criteria include interest and willingness to support the SS during the study period activities, ability to provide any necessary supervision with safety-related mobility activities, and no significant cognitive deficits (as demonstrated by their ability to explain the study purpose and their CP role to the PI during the informed consent discussion). Participants will be requested not to participate in other research studies during the study intervention and evaluation period.

#### Recruitment and retention strategies

Through weekly monitoring of the inpatient and outpatient rehabilitation stroke census, potential participants will be identified by study staff and eligibility criteria will be confirmed based on physician and physical therapy electronic medical records. To broaden recruitment outreach, the PI (SB) and Co-I (TK) will provide stroke information in services to other regional hospitals and community support groups and will partner with other stroke research collaborators in the region to facilitate shared recruitment efforts. The project coordinator will screen interested dyads to confirm eligibility and schedule enrollment. Targeted enrollment rate is two-three dyads per month. To support participant adherence and retention, the project coordinator and PI will be available to respond promptly to any participant concern or issue (via email or phone) and will provide regular check in telephone calls or texts for appointment confirmation reminders. Each study participant receives $50 ($100/dyad) for study participation.

#### Sample size estimation

For Phase II, given the time required to adapt the intervention, the proposal timeline, and preliminary data from our upper extremity intervention, a sample size of 15 dyads is proposed to evaluate feasibility (2-3 dyads/month). An attrition rate of ~8% is projected based on the literature and our previous work [30]; thus, we will enroll 16 dyads to achieve a final sample of 15. Assuming a two-tailed alpha=0.05, we will have 80% power to detect an effect size=0.78 (Cohen, large), and can calculate 95% confidence intervals within 0.64 standard deviations. These data will provide precision to the estimates of mean changes, variability, and effect sizes for key outcomes. Importantly, these data will inform sample size estimation and identify feasibility lessons to guide our planned next step - a randomized clinical trial evaluating the efficacy of CARE-CITE-Gait.

### Intervention

#### CARE-CITE-Gait Intervention

The CARE-CITE-Gait intervention will occur over 4 weeks in the dyad’s home with CP reviewing the CARE-CITE-Gait modules via a web platform (See Table 2). The research interventionist will conduct two-hour, in-home visits at weeks 1 and 4 to develop collaborative goal-setting, and teleconference (phone) check-ins during weeks 2 and 3. At the beginning of each in-home visit, the research interventionist will incorporate clinical assessments of gait function and mobility (5 Times Sit-to-Stand, Timed Up and Go, 10-meter walk; see Table 1 for study schedule and Table 3 for outcome measure description) that mirror lab-based measurements to guide the development of a personalized therapeutic exercise program within the context of each dyad’s unique home environment. In addition to guiding the intervention clinical decision-making for SS and CP goal setting, these additional measurements will provide preliminary data for future validation of non-blinded versus more rigorous blinded in-lab assessments in future study designs.

1) Week 1 Visit - Orientation (2 hours): The research interventionist will meet with CP and SS to review collaborative goals setting, evaluate lower extremity movement, balance, and gait limitations, and identify functional activity preferences and goals. Together, the CP, SS, and interventionist will initiate the development of a progressive therapeutic exercise plan for home-based functional task practice targeting gait and mobility, determine activities or sub-tasks for each goal, develop measurable milestones to accomplish during the treatment period, and create a framework to improve joint responsibilities for self-management skills.

2) CP completes 6 online CARE-CITE modules (15-30-minute sessions each): At the end of the second baseline evaluation, the project coordinator will instruct the CP in the procedures for accessing and viewing the online modules, provide the CARE-CITE-Gait website access password, and guide the CP to review as many modules as possible prior to the first home visit. During Visit 1, the interventionist will review any accessibility issues with the CP and encourage the completion of all 6 modules during the remaining study period. Modules will include demonstration videos and instructive content covering the following areas: principles of functional task practice (i.e., standing and mobility-based activities of daily living such as meal preparation, grooming, household ambulation, or leisure/vocational activities), the adaptation of tasks, and the importance of progressing task challenge and dosage to drive neuroplasticity (i.e., increasing numbers of practice repetitions, walking distance, or balance exercise duration). Examples are provided to address potential SS frustration and improve adherence. Underpinning the content is the concept of autonomy support, with examples of fostering empathy (e.g., the CP joining the SS in walking program), problem-solving (guidance for adaptation of functional activities at home to increase success or challenge), instruction in the use of non-controlling language with role-playing situations and the importance of creating choice in activities. For example, in one vignette to demonstrate problem-solving, a CP interacts with the SS during a standing meal preparation activity and uses non-controlling language to offer choice for task modifications for maintaining balance while reaching for kitchen utensils. The SS becomes frustrated with the challenging task, and the CP demonstrates options for dialogue that foster collaborative problem-solving.

3) Week 2 and 3 telephone check-ins: During weeks 2 and 3 in the 4-week intervention, the research interventionist will complete a structured 10-minute phone call with CP to answer questions, ask the CPs to identify two challenging areas during SS practice activities, and CP opportunities to use autonomy-supportive strategies.

4) Week 4 Home visit (2 hours): At the end of week 4, the same research interventionist will visit the home to review progress with milestones and advancement of activity goals.

#### Standardization

To increase rigor and minimize bias, evaluators will be blinded to study hypotheses/intervention and will be trained in study procedures (e.g., 6-minute walk test) by the PI (SB) and Co-I (TK) with regular assessments of competence every 1.5 months. All efforts will be made to pair the same evaluator with the same dyad for all evaluations to reduce variability. The PI and Co-I will train the research interventionist in delivering the intervention. The PI will provide supervision for the initial in-home visits and phone calls (first 5 dyads) and review the remaining dyad interventions through regular check-ins after each intervention is completed (via email or phone communication). To facilitate protocol adherence, data collection forms for both evaluations and the intervention will be standardized. The laboratory adheres to Good Laboratory Practice[31] principles for clinical research. Working with University Diversity Equity and Inclusion office resources, all study personnel will receive ongoing cultural diversity training throughout the study that will foster education, self-awareness, communication, and community engagement.

### Outcomes

The primary objective of Phase I is to evaluate the content validity and user satisfaction of CARE-CITE-Gait. The primary objective of phase II is to determine the feasibility of CARE-CITE-Gait and our planned study procedures. The secondary objective is to determine the preliminary effects of the CARE-CITE-Gait intervention on both SS and CPs. Measures of CP psychosocial outcomes and SS physical function, gait, and hrQOL will be administered at the rehabilitation hospital stroke research laboratory (in separate rooms) before and after the 4-week intervention (immediately and at 1-month follow-up). See the brief descriptions below and Table 4. Between baselines and after post- and follow-up assessments, SS home and community stepping activity (accelerometry) will be assessed. A select set of measures of SS functional mobility and gait function will also be assessed at intervention home visits to determine the feasibility of assessing outcome measures.

#### Content validity and user satisfaction of CARE-CITE-Gait

In Phase I, we will draw on similar procedures from our previous work [21] evaluating the content validity and user satisfaction of the upper extremity CARE-CITE intervention. To assess CARE-CITE-Gait content validity, content experts (experienced stroke rehabilitation researchers and clinicians) will complete investigator-designed content forms which score the degree to which the content in each module addresses the problem relevance within each content domain. Forms are adapted based on work by Bakas and colleagues. [32] Rehabilitation experts will review the modules for accuracy of content, feasibility, acceptability, problem relevance, and ease of use, using a 5-point Likert response scale (ranging from 1 = strongly disagree to 5 = strongly agree). Nine open-ended reflection questions will gather qualitative feedback regarding most and least helpful modules, areas of concern, areas for improvement, and applicability to clinical practice. Scores will be averaged across each domain for the experts. Experts will review the reflection questions at the end of each module and provide general comments and suggestions for improving content.

To assess CARE-CITE-Gait user satisfaction (usability, acceptability, and overall satisfaction), we will use adapted questions (based on work by Bakas and colleagues [32]) which will be completed by the CP immediately after reviewing of each of the six modules. Satisfaction will be defined as: 1) usefulness of overall content, 2) usefulness of written text, 3) usefulness of videos, 4) ease of use, and 5) acceptability. Each area will be rated using a 5-point Likert type response scale ranging from *1 = strongly disagree* to *5 = strongly agree* and average scores calculated for each subscale as well as a total score. Additionally, time (minutes) to review modules will be recorded by the CP and one open-ended question will be used for general comments or improvement suggestions.

#### Feasibility

To assess the feasibility of the study protocol in Phase II, we will assess participant recruitment and retention, SS and CP adherence to the intervention, and safety (occurrence and type of SS adverse events). The definition and criteria for success are noted in Table 3. We will record the reasons for ineligibility or declining to participate and reasons for study withdrawal after enrollment (obtaining signed consent) in the Consolidated Standards of Reporting Trials (CONSORT) flowchart.

Additional measures of feasibility will include the assessment of CP’s perceptions of CARE-CITE-Gait usability and satisfaction during the one-month intervention period (similar to procedures in Phase I, above). At the end of the one-month follow-up evaluation, CPs will complete the standardized Post Study System Usability Cuestionnaire [33] and a three-section CP Exit Interview questionnaire. Based on post-study participation interviews in similar stroke research [34], this investigator-developed Exit Interview questionnaire was reviewed by experts in caregiving and stroke for content validity. The first section addresses CP confidence in care with sample questions such as: “How confident are you that you can encourage your loved-one when she/he is frustrated with a task?” (15-items, with response scale of 0-100 (0-very uncertain, to 100-very certain)). The second section evaluates the value of participation in the study with questions such as: “Given the time commitment and effort for you to take part in the education project and its effect on your ability to help your loved one, how worthwhile has the participation in the education project been to you personally?” (5 items with a response scale of 1-7 (i.e., 1-not worthwhile to 7-very worthwhile) and two open-ended questions). The third section assesses aspects of helpfulness for the intervention such as: “Given your experience with the education project, how helpful do you feel each of the following aspects (being able to view intervention from your home; homework activities between sessions, the format of using CARE-CITE-Gait website) was for achieving results in your particular case?” with a response scale of 1-7 (i.e., 1-not helpful at all, to 7- very helpful). Two additional open-ended questions ask about helpful areas of the intervention and areas for improvement.

#### Outcomes for Carepartner and Stroke Survivor

Each standardized tool used in Phase II has been tested in the stroke population previously. Table 4 lists CP and SS outcome measures, descriptions, and established reliability and validity. Psychosocial outcomes measured for the CP include family conflict about stroke recovery, strain, autonomy support, and quality of life changes. Outcomes measured for the SS include functional mobility, gait speed, stepping activity, and hrQOL.

#### Additional Assessments

Medical records (for SS) and information questionnaires (SS and CP) will be used to document data about participant characteristics. Information collected for the dyad (both SS and CP) includes demographics (age, gender), marital status, education level, income, work status, any changes in work since stroke, co-morbidities, COVID (past history of testing positive and vaccinations), and current medications. To evaluate adequate participant diversity in recruitment, we will document participants’ self-identified race and ethnicity. From the dyad primary living location zip code, we will determine the Area Deprivation Index (ADI)[35], which characterizes the relative disadvantage of an individual or social network using several US Census indicators of employment, housing, poverty, and education [36]. This collection of data will help guide our recruitment so that we have a diverse and inclusive participant sample based on socioeconomic status as well as race and ethnicity. Specific information gathered for the SS includes the type of stroke, time post-stroke, usual and customary care rehabilitation therapy received (dosage, duration, and frequency), and use of assistive device and/or lower extremity orthotic for mobility. For CP, information collected includes relationship to SS and status of serving as caregiver for other members of family besides SS.

#### Step activity data

To gain information about real-world stepping activity at home and in the community, the SS will be provided with a step activity monitor (Actigraph GT3X+ (Pensacola, FL, USA)), micro-electro-mechanical system (MEMS) based accelerometer and an ambient light sensor in a standardized orientation to be worn on the paretic and non-paretic ankles for up to seven days with intention to at least obtain measures of daily step activity for a minimum of three days. Participants will be provided with verbal and written instructions to don the device upon waking and doff prior to sleeping unless the participant was bathing or taking part in water-based activities. Daily SS stepping activity for the paretic and non-paretic leg before and after the intervention period will be calculated.

### Harms

Adverse events for the SS will be monitored by the intervention therapist each week during the intervention period. Questions related to levels of pain, fatigue, falls or medical appointments will be asked during the home visits and phone call check-ins. The PI will be notified to evaluate and determine if an event is serious or related to the intervention. While no significant risks have been associated with home-based gait and mobility training, the primary risks associated with the intervention involve fatigue, muscle soreness, frustration attempting challenging tasks, and falls. Additional text and videos related to mobility safety (including instruction in the appropriate use of a safety gait belt) have been incorporated into the CARE-CITE-Gait modules and including examples of collaborative discussions for CP and SS to determine safety during mobility training. Safety issues are discussed during home visits as they relate to goal development and home exercise programs. Study evaluators will monitor SS symptoms, heart rate and blood pressure (and oxygen saturation as appropriate) during gait assessments. A gait belt will be used to reduce fall risk during the evaluation and as appropriate during home visits. Any balance or mobility concerns will be relayed to the PI and study interventionist. Risks associated with CP participation reviewing the intervention modules is minimal. Evaluations will include psychosocial assessments evaluating hrQOL and mood while providing care for the stroke survivor. If the study evaluator notices areas of concern (e.g., potential depressive symptoms) the PI will be notified and will recommend to the CP a referral to their primary health care provider for further assessment. Additional community resources will be provided if needed. This process will be explained in the consent form at the initiation of the study. Adverse events will be documented and categorized based on the following University Internal Review Board protocol: serious (death, life-threatening or related inpatient hospitalization) or non-serious; anticipated or unanticipated; related, potentially related, or non-related to the intervention. Description of the event, date, action taken by participant and study personnel (as appropriate), outcomes and any consequent modification of study intervention or procedures will be included in the event description. A Data Safety and Monitoring Board was not established due to minimal CP and SS participation risks. The study team (PI, Co-I, and statistician) will review all adverse events to determine whether any safety concerns warrant trial termination. Safety considerations arising during study evaluations and interventions will be discussed during regular study staff meetings.

### Study Procedures

#### Phase I:

The original CARE-CITE modules were designed as part of a CP-focused web-based intervention aimed to help the CP create a therapeutic home environment while encouraging the SS to use the weaker arm during functional tasks. We (SB, TK, SC, KB) modified the existing upper extremity CARE-CITE intervention to address gait rehabilitation. Collaborating with the University’s Center for Digital Scholarship, the video modules were redesigned with a progressive therapeutic exercise plan for home based functional task practice to improve overall mobility and stepping-related physical activity. Additional content was added to address safety risks/falls (appropriate use of gait belts, supervision, etc.) associated with gait and balance exercises. The gait revisions maintained the core CARE-CITE theoretical framework, which is the concept of autonomy support, with text and video that demonstrate ways to encourage empathy (video examples of discussions of CP with SS acknowledging the difficulty of the task), collaborating on problem-solving (examples of methods to increase or decrease the difficulty of activities together), emphasizing the importance of offering SS choice in activities to practice (examples of joint goal-setting) and ways to provide non-controlling language (scenarios showing controlling vs. non-controlling language).

We will follow similar procedures from our previous work, [21] identifying 6-8 content experts in stroke (rehabilitation researchers and clinicians with at least 5 years of experience) to evaluate the content validity and user satisfaction of the CARE-CITE-Gait intervention. We will identify 3 self-identified CPs of individuals with chronic stroke (> 3 months post-ischemic or hemorrhagic event) to review the CARE-CITE-Gait modules over two weeks and answer module feedback questions. Data gathered about CP user satisfaction and content experts content validity and user satisfaction will guide any additional refinements of intervention before initiation of Phase II study recruitment.

#### Phase II:

Table 1 depicts the schematic diagram of the study schedule and Figure 1 depicts the consort flow chart. The project coordinator will make a clinic appointment at Emory Rehabilitation Hospital for interested participants. If screening criteria are met, written informed consent will be obtained from the CP and SS by the study PI (SB) or project coordinator (HS). Medical clearance from the SS physician will be obtained before study participation. Double baseline data collection visits will be conducted in the clinic/lab, one week apart, by the research evaluator (licensed physical therapist) blinded to the content of CARE-CITE-Gait intervention. Following the second baseline data collection, the research interventionist (licensed physical therapist) will schedule dyads for the Week 1 (orientation) and Week 4 home visits and complete Week 2 and 3 phone calls. The project coordinator will schedule all dyad data collection appointments. All dyads return to the clinic for the post-test, and 1-month follow-up visits. Accelerometers (wearable sensor) data will be gathered between the first and second baseline assessments (pre-training), and after the post, and 1-month follow-up assessments. Accelerometers will be worn for all activities for a minimum of 3 days up to a target of 7 days, except for water-based activities. We will provide SS with wearable sensors and instructions regarding compliance and data logging for sensor-based activity data.

### Data management and analysis

REDCap (Research Electronic Data Capture)[37] electronic database will be used to store the quantitative data. REDCAP is a secure (compliant with United States healthcare confidentiality legislation requirements), web-based application designed to support data capture for research studies, providing 1) an intuitive interface for validated data entry; 2) audit trails for tracking data manipulation and export procedures; 3) automated export procedures for seamless data downloads to common statistical packages; and 4) procedures for importing data from external sources. The project coordinator will be trained in REDCap processes and will complete all data entry. Data will be double checked for verification by a second REDCap trained research assistant. REDCap creates a comma separated value file and SAS program to create an analytic dataset. These files and all resulting datasets, programs, and results are stored on a HIPAA compliant Rollins School of Public Health sever.

Standard data cleaning, identification of missing data, and internal consistency reliability for standardized scales will be completed. We will investigate whether missing data is related to treatment and baseline factors but do not anticipate missing data being a limitation if lost-to-follow-up rates in this new clinical trial are similar to other stroke studies performed in the PI (SB) and Co-I (TK) labs. Primary quantitative statistical analyses will be performed by the study statistician (GC) using SAS statistics for Windows (Version 9.4) and accelerometry data analysis (SS stepping activity) will be performed by lead research assistant (RS) using ActiLife software (Version 6.13.4). Descriptive statistics (e.g., frequencies, means, ranges, standard deviations) will be calculated for all relevant variables, as well as to identify unusual or suspect values requiring review and confirmation. Additional descriptive statistics will be calculated for feasibility measures of recruitment rate, retention, and CP usability and satisfaction with CARE-CITE-Gait (described under Feasibility section). To determine estimates of variability (for postulating effect sizes to design next phase clinical trials, the mean and median changes from baseline to one month and standard errors of the means will be calculated. To provide preliminary information about possible feasibility study intervention effects, confidence intervals for the difference in mean changes for major study variables will be reported as descriptive statistics. Graphical methods will be used to investigate outliers and investigate the consistency of the changes over time. Estimates of inter-correlations among the study variables will be calculated to gain insight into potential variables that could influence response to the intervention and guide the design of future studies. To understand potential relationships and possible confounding factors we will examine SS co-morbidities (e.g., history of testing positive for COVID) and the biological variables of CP age, sex, gender and relationship of CP to SS (e.g. spouse, adult child). The second phase of the analysis will include one-way repeated measures analysis of variance (ANOVA) to evaluate differences between baseline, post, and follow-up timepoints. Tukey’s pairwise comparisons will be used to determine which time points are different if necessary. Level of significance of 0.05 will be used. No adjustments will be used to control for multiple study variables.

### Auditing

All research records will be available for review by authorized representatives of the Foundation for Physical Therapy Research, regulatory agencies and the University Institutional Review Board to monitor study safety, progress, and procedures for quality assurance.

### Dissemination

Dissemination of study results to academic communities will occur through peer-reviewed manuscripts and regional and national conference presentations. The study team will work collaboratively with the funding agency to share results based on agency requirements. The PI will meet regularly with the study team to revise the proposed dissemination plan and discuss authorship guidelines. For non-academic communities, the PI will schedule presentations with community stroke groups and therapy clinics involved in study recruitment. A lay summary of study findings will be created for distribution based upon participant requests.

## Discussion

This protocol describes the methodology to evaluate the feasibility of a CP-integrated telehealth gait rehabilitation program during stroke recovery. The study findings will provide valuable insights regarding innovative home-based rehabilitation that engages CPs and support a future study testing the efficacy of CARE-CITE-Gait intervention. Results will provide important preliminary estimates of efficacy and components of variability as well as inform future randomized clinical trial sample size power calculations. Data gathered regarding recruitment, retention, and adherence rates, adverse events, outcome measure appropriateness and participant exit interviews will inform the feasibility and justification of a definitive trial of CARE-CITE-Gait. Our proposal will lay foundations for several research trajectories with the long-term goal of developing more personalized, precise, and efficacious family-focused rehabilitation interventions.

## Trial Status

This trial was registered on clinicaltrials.gov (NCT 05257928) on March 25, 2022. Recruitment of participants was initiated on May 18, 2022. To date, we have recruited 90% of the participants.

## Figures and Tables

**Table 1:** CARE-CITE-Gait study schedule.

	STUDY PERIOD								
				CARE-CITE-Gait Intervention					
	Screen	Baseline 1	Baseline 2	Week 1	Week 2	Week 3	Week 4	Post Intervention Evaluation	1-month Follow-up Evaluation
Eligibility screen	PC								
Informed consent		PI							
Medical history (including COVID questionnaire)	x	x	x (updates)					x (updates)	x (updates)
Demographics		x							
SS Vitals (BP, HR, O_2_ saturation)		x	x	*			*	x	x
SS 10-meter walk test		x	x	*			*	x	x
SS 6-minute walk test		x	x					x	x
SS 5STS		x	x	*			*	x	x
SS TUG		x	x	*			*	x	x
SS SIS		x						x	x
SS FCCQ-SS		x	x					x	x
SS ABC		x	x	*			*	x	x
SS Accelerometry (3-7 days)		x	x					x	x
CP CSI		x	x					x	x
CP FCCS		x	x					x	x
CP BCOS		x	x					x	x
CP FCCQ-CP		x	x					x	x
CP CCG Module Feedback questions				CP			CP		
CP CCG Module Reflection questions				CP			CP		
CP PSSUQ									x
CP Exit Interview									x
Adverse events monitoring		*							*

SS-stroke survivor, CP-carepartner, CCG-CARE-CITE-Gait, BP-blood pressure, HR-heart rate, O_2_-oxygen saturation, 5STS-5-times Sit to stand, TUG-Timed up and go test, SIS-Stroke Impact Scale, FCCQ-SS-Family Care Climate Questionnaire for stroke survivor, ABC-Activities-Specific Balance Confidence Scale, CSI-Caregiver Strain Index, BCOS-Bakas Caregiving Outcomes Scale, FCCS-Family Caregiver Conflict around Stroke Recovery Scale, FCCQ-CP - Family Care Climate Questionnaire for carepartner, PSSUQ-Post Study System Usability Questionnaire PC-completed by project coordinator, PI-completed by PI, x-completed by evaluator

* completed by intervention therapist

**Table 2.** Content of the CARE-CITE-Gait Intervention for the Carepartner

Modules	Content
Overview of Modules Structure	Each module has multimedia to provide the purpose, educational content, and/or illustrate examples of the topics discussed. Concluding each module, 4-5 reflection questions are provided to allow for application of content and 7 feedback questions are provided to gather information on ease-of-use, acceptability, and usefulness of modules.
Module I: Introduction to CARE-CITE-Gait	Describes CARE-CITE Gait project and defines the roles of the CP and SS and summarizes the modules. Welcome survey provided for CP to complete with research interventionist for practice using the website and answering questionnaires.
Module II: Introduction to Carepartner and Collaborative Integrated Therapy – CARE-CITE	Overview of goal setting (providing examples in the areas of household activities of daily living, leisure, and work-related, and collaborative activities), home diary (to record activities and difficulty level), and review of safety measures and behavior contract (use of gait belt, agreement between stroke survivor and carepartner on individual and collaborative activities). Two videos showing examples of practicing activities of daily living to improve balance and gait, image examples of a completed home diary and behavior contract, and seven videos of conversations around safety and considerations.
Module III: Practice and Goal Setting	Review of role of practice in promoting neuroplasticity and recovery after stroke. Discussion of collaborative problem-solving to accomplish tasks, maintaining appropriate challenge threshold, both reducing task complexity when a task is too difficult and increasing challenge when the task is easily mastered. Two to seven video clips capture each of the six themes of practice.
Module IV: Autonomy Support – Creating Partnerships	Cultivating an autonomy supportive environment with empathy, problem-solving through tasks in the home setting, use of non-controlling language, and offering choice. Recognize challenges and explore ways to improve communication (avoid controlling language such as “you should exercise,” or “you have to do this”). Eight video clips illustrate understanding another’s viewpoint, using problem-solving strategies, providing rationale, and providing choice during gait and mobility exercises.
Module V: Taking Care of Yourself as a Carepartner	CP self-care – recognizing demands of caregiving role, strategies for stress reduction, opportunities for self-care activities and community resources (only text).
Module VI: Reflections	Six videos (limited text) of stroke survivors and CP reflecting on rehabilitation and recovery. Encouraging CP reflection on his/her role in recovery of the individual with stroke.

**Table 3:** CARE-CITE-Gait Study Feasibility Outcomes

Outcome	Definition
Recruitment	Recruitment rates will be the percentage of those participants enrolled and randomized from those screened. Recruitment will be deemed feasible if the target enrollment of 15 dyads (2-3 dyads per month) is reached during the study timeframe.
Retention	Retention of participants will be the percentage of dropouts. An acceptable retention rate will be 85% of enrolled participants for completion of post-evaluation (80% for one-month follow-up).
Intervention Adherence*	CP adherence will be the number of modules reviewed (6 total modules) as indicated by the reflection questions completed at the end of each section. Criteria for CP adherence will be a minimum completion of 5 of the 6 modules. *Study staff are electronically notified of module completion in real-time which allows for reminders to be sent to participants to review modules as needed*. Criteria for CP and SS adherence will be >3.5 hours (for two home visits) and CP completion of week 2 and 3 phone calls.
Safety	Number and type of adverse events (serious vs. non-serious; related or possibly related to CARE-CITE-Gait

* Adherence to the intervention will be evaluated only in the participants completing a baseline and a post-intervention evaluation. SS-stroke survivor; CP-carepartner

**Table 4.** Outcome measures collected at baseline, post-intervention, and 1-month follow-up

Variable	Measure	Description	Reliability/Validity
SS Gait Speed*	10-meter walk test^32^	Time to complete a standardized overground distance; correlated with functional ambulation categories	Excellent test-retest (ICC>0.95),[38] intra- and inter-rater reliability (ICCs>0.87); established construct [39]and criterion[40] validity in stroke
SS Endurance	6-minute walk test^33^	Distance walked in 6-minutes; measure of aerobic capacity and long-distance walking function	SEM, MDC, and MCID published in older adults and stroke; excellent reliability (ICC>0.99); validity established in stroke[39, 41, 42]
SS Dynamic Balance and Functional Mobility*	5-times Sit to stand (5STS)^34^ Timed up and go test (TUG)^35^	Timed tests of functionally relevant lower limb activities (sit to stand, walking, turning)	5STS - Criterion validity with leg muscle strength in stroke; discriminates stroke versus able-bodied;[43, 44] [45] TUG – SEM, MDC, reliability, validity published in stroke[39, 46, 47]
SS Quality of Life	Stroke Impact Scale (SIS)	59-items, 8 domains function	Test-retest reliability ICC = 0.70 to 0.92; Internal consistency alpha coefficient of 0.83-0.90 [48]
SS Mobility Confidence*	Activities-Specific Balance Confidence Scale (ABC)	16 items, self-report of balance confidence with balance-related activities	Adequate to excellent test-retest reliability for total score ICC=0.85), excellent internal consistency[49, 50]
SS Average stepping activity**	Wearable sensors (Actigraph^38^)	Actigraph GT3X+ *(Actigraph Pensacola, Florida, USA)* triaxial accelerometer; raw acceleration data are converted into activity counts per minute.	Step activity monitors have been shown to have excellent test-retest reliability on 3-day monitoring (ICC>0.9); criterion validity published in stroke [51] [52–54]
CP/SS Autonomy Support Environment	Family Care Climate Questionnaire FCCQ-CP/FCCQ-SS	14-item, Likert-type scale. Higher scores/higher autonomy support perception	Internal consistency >. 70; Construct validity supported- higher FCCQ-SS scores related to SS lower perception of criticism, higher family emotional involvement-higher satisfaction with family support (p ≤ .05)[55]
CP Strain	Caregiver Strain Index - CSI (modified)	13-item questionnaire, binary yes/no; higher score/higher strain	Good reproducibility and validity in stroke carepartners, Cronbach’s alpha of .83 [56–59]
CP Family Conflict	Family Caregiver Conflict Scale (FCCS) about Stroke Recovery	15-item, Likert-type scale Higher scores/higher conflict	Established content/construct validity in stroke CP; reliability Cronbach’s alpha of .93[60]
CP Well-being related to Caregiving	Bakas Caregiving Outcome Scale (BCOS)	15-items; 7 point scale; higher scores/more positive caregiving outcomes	Satisfactory reliability and validity in stroke carepartner, Cronbach’s alpha of .90 [61]
CARE-CITE-Gait Usability							
CP experience in CARE-CITE	Exit Interview questionnaire	Three sections assessing confidence in care, value of participation and aspects of CARE-CITe	Interview guide will be reviewed by content and qualitative experts prior to use.
CP satisfaction with CARE-CITE	Feedback forms at end of CARE-CITE modules	5-items, Likert-type scale; higher scores/higher satisfaction	Interview guide will be reviewed by content and qualitative experts prior to use.
CP experience using CARE-CITE	Post Study System Usability Questionnaire (PSSUQ)	19-item, Likert-type scale. Lower scores/greater usability of instrument	Established reliability and validity[33]

SS-stroke survivor, CP-carepartner, CCG-CARE-CITE-Gait, BP-blood pressure, HR-heart rate, O_2_ – oxygen saturation, 5STS-5-times Sit to stand, TUG-Timed up and go test, SIS-Stroke Impact Scale, FCCQ-SS-Family Care Climate Questionnaire for stroke survivor, ABC-Activities-Specific Balance Confidence Scale, CSI-Caregiver Strain Index, BCOS-Bakas Caregiving Outcomes Scale, FCCS-Family Caregiver Conflict around Stroke Recovery Scale, FCCQ-CP - Family Care Climate Questionnaire for carepartner, PSSUQ-Post Study System Usability Questionnaire PC

* additional administration during home visit week 1 and week 4

** accelerometers worn at home for 7 days

## Data Availability

The datasets generated and/or analyzed during the current study are not publicly available due to some data containing information that could compromise research participant privacy/consent but are available from the corresponding author [sb] on reasonable request.

## Abbreviations

CARE-CITE-Gait: Carepartner and Collaborative Integrated Therapy-Gait
CP: Carepartner
SS: Stroke Survivor
FCCS: Family Caregiver Conflict Scale about Stroke Recovery
FCCQ: Family Care Climate Questionnaire
CSI: Caregiver Strain Index
BCOS: Bakas Caregiving Outcomes Scale
SIS: Stroke Impact Scale
5STS: 5-times Sit to stand
TUG: Timed up and go test
SIS: Stroke Impact Scale
ABC: Activities-Specific Balance Confidence Scale
PI: Principal Investigator
Co-I: Co-Investigator
